# Supplementary material for: Dose-dependent response to infection with SARS-CoV-2 in the ferret model and evidence of protective immunity
Source: Nat Commun. 2021 Jan 4;12:81. doi: 10.1038/s41467-020-20439-y (PMC7782478; doi:10.1038/s41467-020-20439-y)
Supplement: Supplementary file 1 — Supplementary information [file 41467_2020_20439_MOESM1_ESM.pdf]

Supplementary Information

Dose-dependent response to infection with SARS-CoV-2 in the ferret model and evidence of protective immunity

Detection of Viral RNA

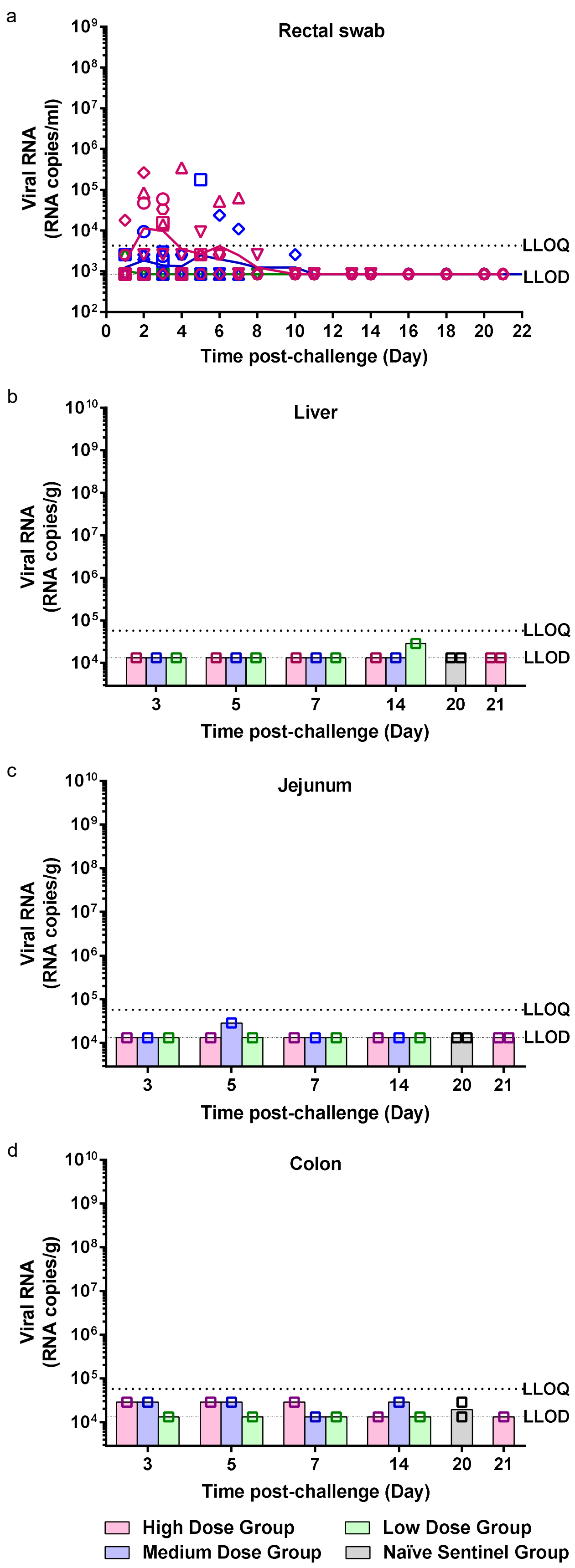

Supplementary Figure 1 Detection of viral RNA Rectal swabs were collected at days 1 to 8, 10, 11, 13, 14, 16, 18 & 20 pc for all virus challenged groups. Viral genomic RNA was quantified by RT-qPCR at all timepoints. No viral RNA was detected in any samples taken from the naïve sentinel ferrets. (**a**) Rectal swabs. Viral genomic RNA was quantified by RT-qPCR in the (**b**) liver, (**c**) jejunum and (**d**) colon when collected at euthanasia timepoints (days 3, 5, 7, 14, 20 and 21pc) for all groups. The dashed horizontal lines show the lower limit of quantification (LLOQ) and the lower limit of detection (LLOD).

Antibody immune response of ferrets challenged with SARS-CoV-2

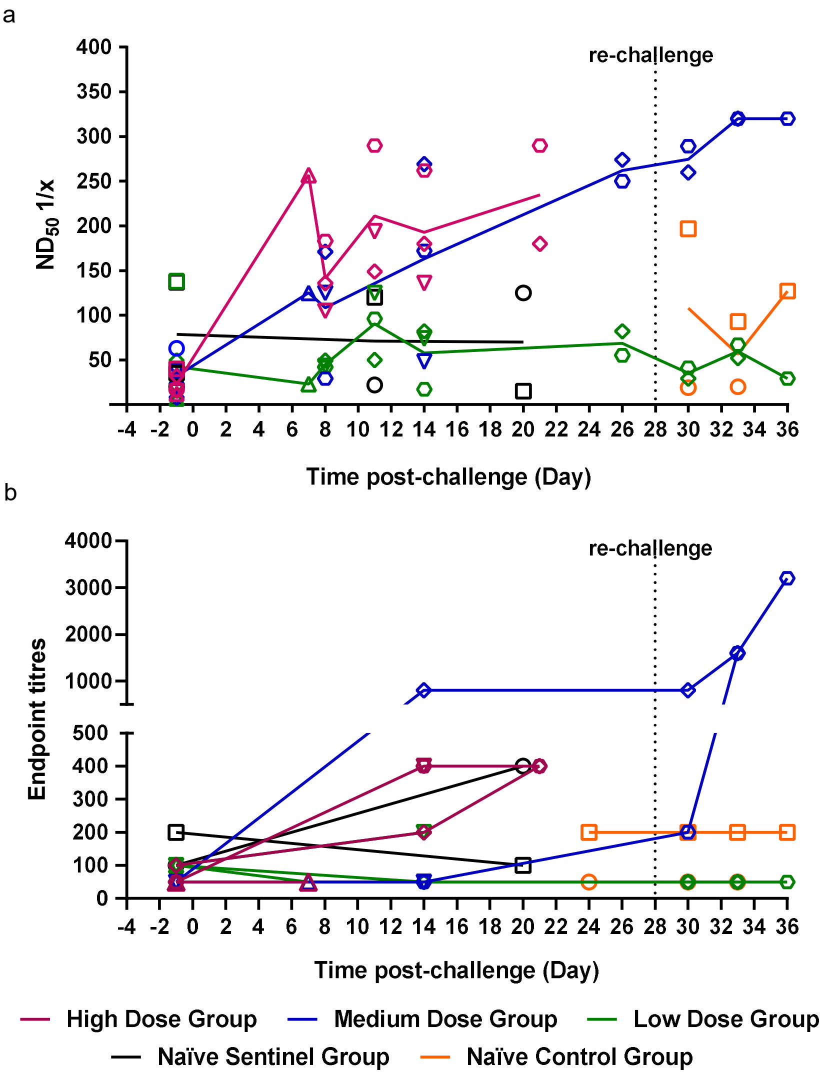

Supplementary Figure 2 Antibody immune response of ferrets challenged with SARS-CoV-2 (**a**) Neutralising antibodies in serum measured by Plaque reduction neutralisation test ($PRNT_{50}$). Serum neutralisation titres as reciprocal highest dilution resulting in an infection reduction of >50% in samples ($PRNT_{50}$) pre-challenge (-1) and at 7, 11, 14, 20, 21, 26 days post challenge and days 3, 5 and 8 post re-challenge in ferrets. Points show values for individual ferrets, lines show group means. Neutralising antibodies were observed at 7 dpc, increasing from 11dpc onwards, (**b**) SARS-CoV-2-specific IgG antibodies measured by ELISA in naïve and SARS-CoV-2 infected ferrets**.** Spike specific IgG antibodies measured in sera of ferrets. Sera were analysed from uninfected ferrets (day -1, 0 and 24) or 7, 14, 20 and 21 days post challenge and 30, 33 and 36 days post challenge. Points and lines show endpoint titre values for individual ferrets.

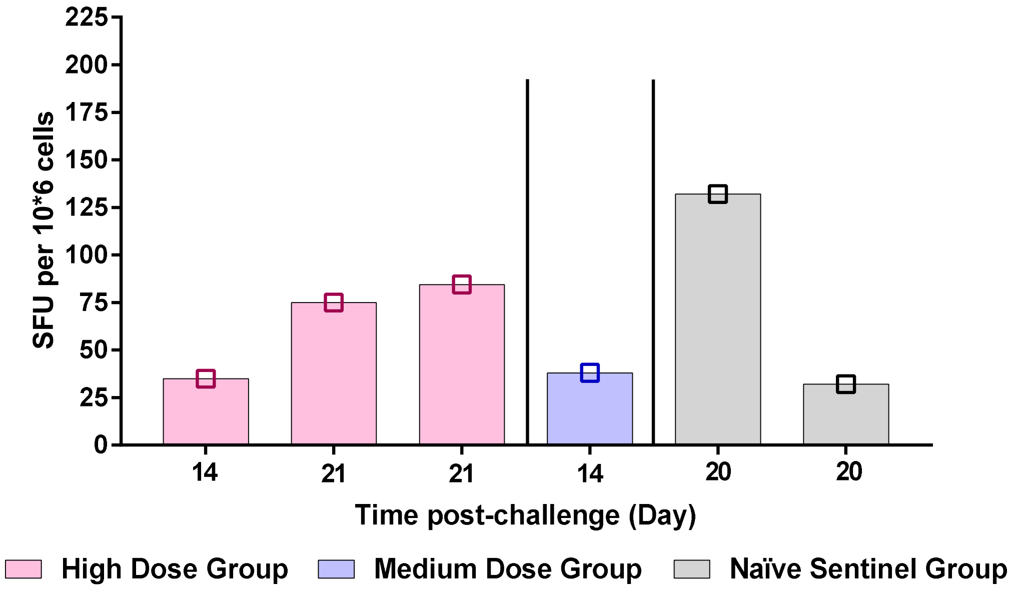

Supplementary Figure 3. Cellular immune responses of ferrets challenged with SARS-CoV-2 Lung MNCs were collected from animals (n= 1) at day 14, 21 and 20 pc. SARS-CoV-2 specific IFN-γ responses were seen in all ferrets, including the two naïve sentinel ferrets. The values measured for each ferret are plotted as spot forming units (SFU) per million cells.

Viral RNA Shedding from Re-challenged Ferrets

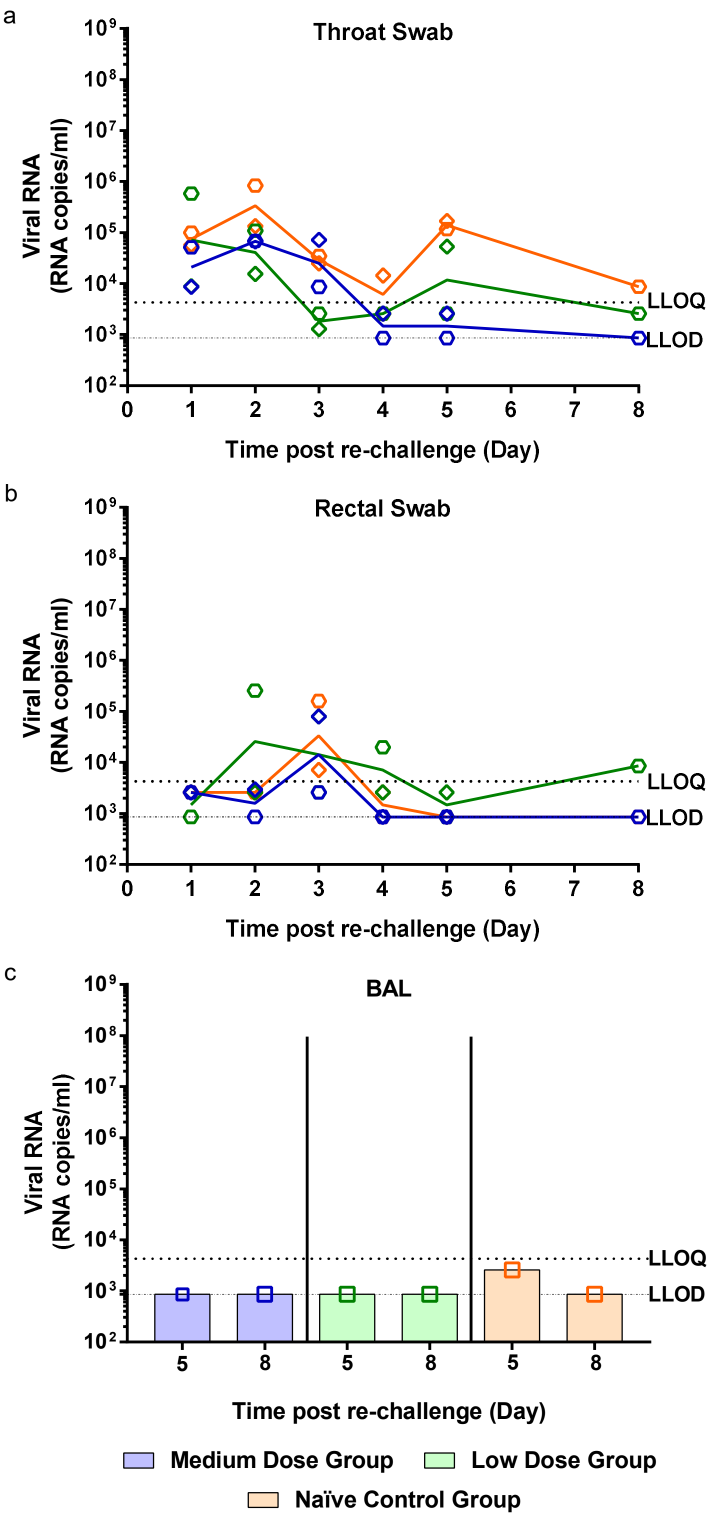

Supplementary Figure 4. Viral RNA Shedding from re-challenged ferrets. Swabs were collected at 1 to 5 & 8 post re-challenge for all virus challenged groups. Viral RNA was quantified by RT-qPCR. (**a**) Throat swabs (**b**) rectal swabs (**c**) Bronchoalveolar lavage collected at necropsy (numbers indicate day post re-challenge the ferret was euthanised). Points show values for individual animals, lines show group geometric means. The dashed horizontal lines show the lower limit of quantification (LLOQ) and the lower limit of detection (LLOD).

Viral Subgenomic RNA Shedding from Re-challenged Ferrets

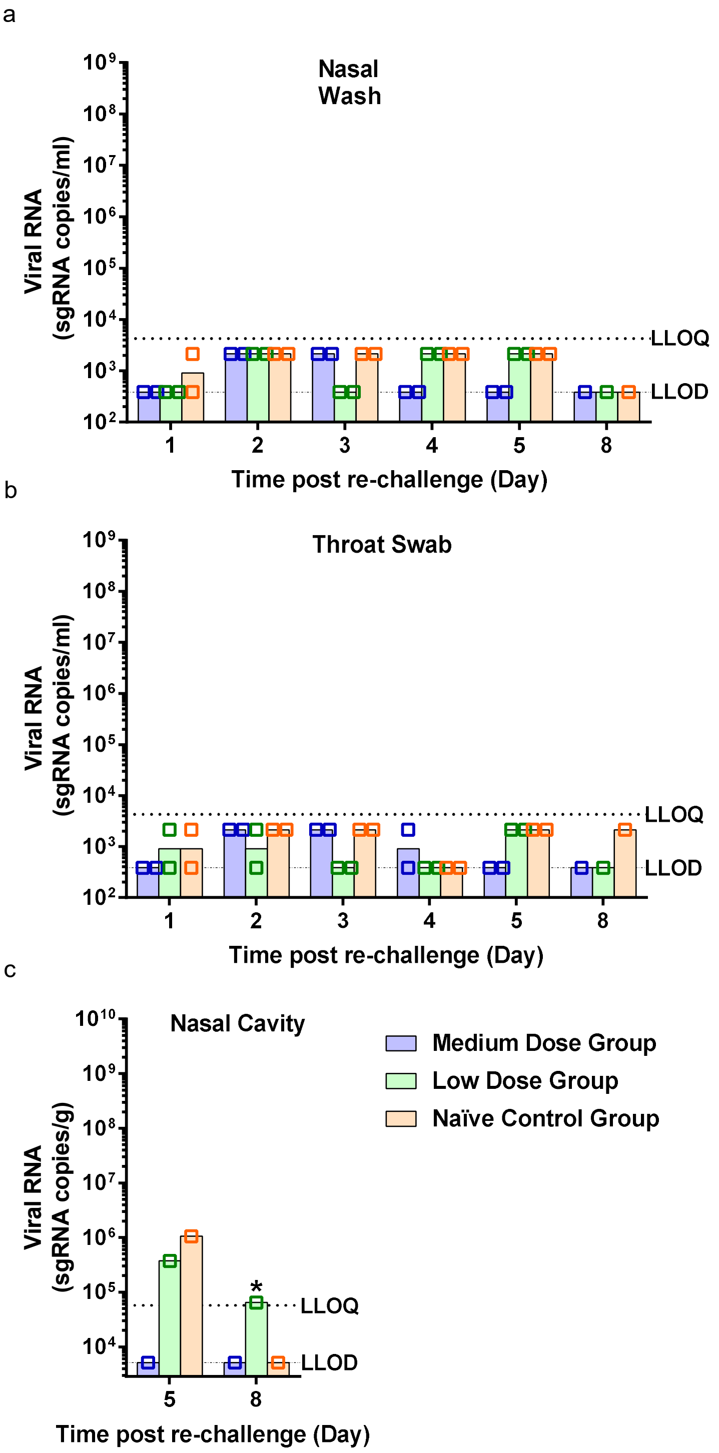

Supplementary Figure 5. Viral subgenomic RNA shedding from re-challenged ferrets. Nasal washes and throat swabs were collected at 1 to 5 & 8 post re-challenge for all virus challenged groups. (**a**) Nasal wash (**b**) throat swabs (**c**) Nasal cavity collected at necropsy (numbers indicate day post re-challenge the ferret was euthanised). *Sample RNA undetected by sgPCR but only 2.4 mg homogenised (LLOD $6.43x10^4$ for this sample only). Points show values for individual animals, lines show group geometric means. The dashed horizontal lines show the lower limit of quantification (LLOQ) and the lower limit of detection (LLOD).

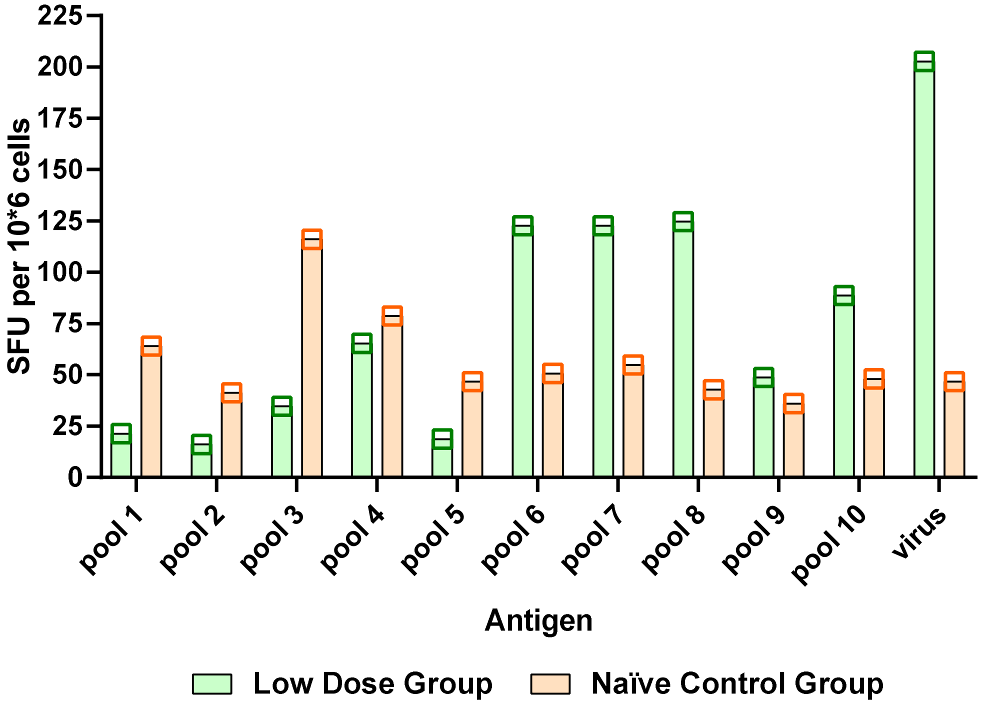

Supplementary Figure 6. Cellular immune responses of ferrets re challenged with SARS-CoV-2. Lung MNCs were collected from animals (n= 1) at day 36 pc (day 8 following re-challenge). Lung MNCs were stimulated with individual peptide pools spanning the spike and whole live SARS-CoV-2 virus. SARS-CoV-2 specific IFN-γ responses were seen in both infected ferrets. The values measured for each ferret are plotted as spot forming units (SFU) per million cells.

Supplementary Table 1. Sequences of the primers and probes

**Name**	**Sequence**
2019-nCoV_N1-forward	5’ GACCCCAAAATCAGCGAAAT 3’
2019-nCoV_N1-reverse	5’ TCTGGTTACTGCCAGTTGAATCTG 3’
2019-nCoV_N1-probe	5’ FAM-ACCCCGCATTACGTTTGGTGGACC-BHQ1 3’
2019-nCoV_sgE-forward	5’ CGATCTCTTGTAGATCTGTTCTC 3’
2019-nCoV_ sgE-reverse	5’ ATATTGCAGCAGTACGCACACA 3’
2019-nCoV_ sgE-probe	5’ FAM-ACACTAGCCATCCTTACTGCGCTTCG- BHQ1 3’

Supplementary Table 1. Sequences of the primers and probes. Name and sequence of primers and probes utilised in RT-qPCR and sub-genomic PCR methods.
