# Supplementary material for: Dose-dependent response to infection with SARS-CoV-2 in the ferret model and evidence of protective immunity
Source: Nat Commun. 2021 Jan 4;12:81. doi: 10.1038/s41467-020-20439-y (PMC7782478; doi:10.1038/s41467-020-20439-y)
Supplement: Supplementary file 3 — Reporting summary [file 41467_2020_20439_MOESM3_ESM.pdf]

nature research

Corresponding author(s): MIles Carroll

Last updated by author(s): 2020/11/02

Reporting Summary

Nature Research wishes to improve the reproducibility of the work that we publish. This form provides structure for consistency and transparency in reporting. For further information on Nature Research policies, see our Editorial Policies and the Editorial Policy Checklist.

Statistics

For all statistical analyses, confirm that the following items are present in the figure legend, table legend, main text, or Methods section.

n/a	Confirmed	
☐	☒	The exact sample size (n) for each experimental group/condition, given as a discrete number and unit of measurement
☐	☒	A statement on whether measurements were taken from distinct samples or whether the same sample was measured repeatedly
☐	☒	The statistical test(s) used AND whether they are one- or two-sided<br>*Only common tests should be described solely by name; describe more complex techniques in the Methods section.*
☒	☐	A description of all covariates tested
☒	☐	A description of any assumptions or corrections, such as tests of normality and adjustment for multiple comparisons
☐	☒	A full description of the statistical parameters including central tendency (e.g. means) or other basic estimates (e.g. regression coefficient) AND variation (e.g. standard deviation) or associated estimates of uncertainty (e.g. confidence intervals)
☐	☒	For null hypothesis testing, the test statistic (e.g. F, t, r) with confidence intervals, effect sizes, degrees of freedom and P value noted<br>*Give P values as exact values whenever suitable.*
☒	☐	For Bayesian analysis, information on the choice of priors and Markov chain Monte Carlo settings
☒	☐	For hierarchical and complex designs, identification of the appropriate level for tests and full reporting of outcomes
☒	☐	Estimates of effect sizes (e.g. Cohen's d, Pearson's r), indicating how they were calculated

Our web collection on statistics for biologists contains articles on many of the points above.

Software and code

Policy information about availability of computer code

Data collection	No software was used for data collection.
Data analysis	GraphPad Prism 8.0, ImmunoSpot 5.0, QuantStudio™ 7

For manuscripts utilizing custom algorithms or software that are central to the research but not yet described in published literature, software must be made available to editors and reviewers. We strongly encourage code deposition in a community repository (e.g. GitHub). See the Nature Research guidelines for submitting code & software for further information.

Data

Policy information about availability of data

All manuscripts must include a data availability statement. This statement should provide the following information, where applicable:

- Accession codes, unique identifiers, or web links for publicly available datasets
- A list of figures that have associated raw data
- A description of any restrictions on data availability

SARS-CoV-2 N ORF (accession number NC_045512.2)
SARS-CoV-2 E ORF (accession number NC_045512.2)

Field-specific reporting

Please select the one below that is the best fit for your research. If you are not sure, read the appropriate sections before making your selection.

- [x] Life sciences
- [ ] Behavioural & social sciences
- [ ] Ecological, evolutionary & environmental sciences

For a reference copy of the document with all sections, see nature.com/documents/nr-reporting-summary-flat.pdf

Life sciences study design

All studies must disclose on these points even when the disclosure is negative.

Sample size	No sample size calculation was performed. As this was an initial pilot dose-ranging investigation into SARS-CoV-2 infection in ferrets, 6 animals per challenge group was selected. As per several other published early reports minimal numbers of animals were used for sequential culls. We believe it provides important information regarding the ferret model of SARS-CoV-2 going forward.
Data exclusions	No data were excluded from the analysis.
Replication	Animal experiments were not replicated due to ethical considerations when using animal models.<br>Samples for RT-qPCR and sgPCR were assayed in duplicate against a standard curve in triplicate.
Randomization	Animals were randomly allocated to challenge groups according to their social housing upon arrival.
Blinding	Blinding was used in analysis of the histological slides. Numbers were randomly allocated to slides. Examination and analysis was carried out blinded and at random by independent pathologists.

Reporting for specific materials, systems and methods

We require information from authors about some types of materials, experimental systems and methods used in many studies. Here, indicate whether each material, system or method listed is relevant to your study. If you are not sure if a list item applies to your research, read the appropriate section before selecting a response.

Materials & experimental systems

n/a	Involved in the study	
☐	☒	Antibodies
☐	☒	Eukaryotic cell lines
☒	☐	Palaeontology and archaeology
☐	☒	Animals and other organisms
☒	☐	Human research participants
☒	☐	Clinical data
☒	☐	Dual use research of concern

Methods

n/a	Involved in the study	
☒	☐	ChIP-seq
☒	☐	Flow cytometry
☒	☐	MRI-based neuroimaging

Antibodies

Antibodies used	Goat anti-ferret IgG conjugated to HRP (Novus Biologics, NB7224)
Validation	By immunoelectrophoresis and ELISA this Goat anti-Ferret IgG (H+L) Secondary Antibody [HRP] reacts specifically with ferret IgG and with light chains common to other ferret immunoglobulins. This may cross react with IgG from other species.

Eukaryotic cell lines

Policy information about cell lines

Cell line source(s)	Vero/hSLAM cells [ECACC 04091501], Vero/E6 cells [ECACC 85020206]
Authentication	Cell lines were obtained from the European Collection of Authenticated Cell Cultures (ECACC) PHE, Porton Down, UK.
Mycoplasma contamination	All cell lines used were confirmed negative for mycoplasm.
Commonly misidentified lines<br>(See ICLAC register)	No commonly misidentified cells lines were used in this study.

Animals and other organisms

Policy information about studies involving animals; ARRIVE guidelines recommended for reporting animal research

Laboratory animals	Ferret (Mustela putorius furo), female, 7 months of age
Wild animals	No wild animals were used during this study.
Field-collected samples	No field-collected samples were used during this study.
Ethics oversight	All experimental work was conducted under the authority of a UK Home Office approved project licence (PDC57C033) that had been subject to local ethical review at PHE Porton Down by the Animal Welfare and Ethical Review Body (AWERB) as required by the Home Office Animals (Scientific Procedures) Act 1986.<br><br>The study protocol was approved by ethical review at PHE Porton Down by the Animal Welfare and Ethical Review Body (AWERB).

Note that full information on the approval of the study protocol must also be provided in the manuscript.
